# Newborn screening for lysosomal disorders in Brazil: A pilot study using customized fluorimetric assays

**DOI:** 10.1590/1678-4685-GMB-2018-0334

**Published:** 2020-05-29

**Authors:** Fernanda Bender, Maira G. Burin, Kristiane M. Tirelli, Fernanda Medeiros, Fernanda Hendges de Bitencourt, Gabriel Civallero, Francyne Kubaski, Heydy Bravo, Antoine Daher, Vanessa Carnier, José F. S. Franco, Roberto Giugliani

**Affiliations:** Hospital de Clínicas de Porto Alegre, Medical Genetics Service, Porto Alegre, RS, Brazil.; Universidade Federal do Rio Grande do Sul (UFRGS), Programa de Pós-Graduação em Médicas: Ciências Médicas, Porto Alegre, RS, Brazil.; Instituto Nacional de Genética Médica Populacional (INAGEMP), Porto Alegre, RS, Brazil.; Universidade Federal do Rio Grande do Sul (UFRGS), Departamento de Genética, Porto Alegre, RS, Brazil.; Casa Hunter, São Paulo, SP, Brazil.; Pontifícia Universidade Católica de Campinas, Hospital e Maternidade Celso Pierro, Campinas, SP, Brazil.

**Keywords:** Inborn errors of metabolism, newborn screening, enzymes

## Abstract

Lysosomal storage disorders (LSDs) are a group of genetic disorders characterized by deficiency of specific lysosomal enzymes. In general, patients are clinically normal at birth, and progressively develop severe signs and symptoms. Diagnosis is usually made several years after onset of manifestations, preventing patients to have the benefits of the early treatment. Newborn screening programs are being considered for LSDs to allow early diagnosis and treatment. The present study evaluated the feasibility of a customized screening approach based on modified fluorometric assays with reduced amounts of reagents, substrates and samples for: mucopolysaccharidosis (MPS) type I (MPS I), MPS VI, Fabry, Gaucher, and Pompe diseases. We also evaluated the advantages of including blood chitotriosidase and urinary glycosaminoglycans in the protocol. By the measurement of the specific disease-associated enzymes (plus blood chitotriosidase and urinary glycosaminoglycans) we analyzed 834 de-identified DBS of unselected newborns. No positive case was detected, and the false-positive rates were low. Taking into consideration the limitations of this methodology, we believe that, after defining proper cutoffs, it could be a viable alternative to provide NBS for LSDs by laboratories that may not be able to afford the commercial methods available.

## Introduction

Lysosomal storage disorders (LSDs) are a group of inborn errors of metabolism (IEM) that result from the deficiency of specific hydrolases, protein activators or transport proteins, leading to an accumulation of undegraded substrates in lysosomes leading to biochemical changes and even cell death (Tager *et al.*, 1984).

LSDs include over 50 genetic disorders with specific characteristics (Bellettato and Scarpa, 2010). Although, they are considered individually rare, when combined they are estimated to occur in 1:7,700 live births (Meikle *et al.*, 1999). The incidence of LSDs in Brazil is still not clear. A study reported that LSD corresponded to 60% of the IEM diagnosed from 1982 to 2015 at a reference laboratory in Brazil (Giugliani *et al.*, 2017).

Newborn screening (NBS) allows the early diagnosis of several congenital disorders that are mainly asymptomatic at the newborn period. The diagnosis allows early treatment in order to at least slow down the disease progression (http://portalms.saude.gov.br/acoes-e-programas/programa-nacional-da-triagem-neonatal). Ideally, the tests included in an NBS program should be based in a reliable methodology and should have the possibility of multiplexing.

In Brazil, there are few records of NBS programs involving LSDs. In Porto Alegre in southern Brazil, the private laboratory CTN screened 10,527 babies for Fabry, Gaucher, Pompe, and MPS I. All initially positive cases were further studied until a conclusion was made, and no diagnosis was confirmed (Camargo Neto *et al.*, 2018). In Monte Santo, Bahia state, where there is a high incidence of MPS VI (approximately 1:5,000), a common mutation (p.His178Leu) was found in homozygosis in 13 patients and a high carrier frequency (40%) was observed (Costa-Motta *et al.*, 2011), confirming that a founder effect and endogamy play a major role in this case. A newborn screening pilot program for MPS VI (based on the detection of the common mutation) was introduced in Monte Santo and already tested over 5,000 babies (Bender, 2011).

Here, our aim was to test customized fluorometric methods for assaying five lysosomal enzymes in screens for MPS I, MPS VI, Fabry, Gaucher, and Pompe diseases, plus assays of biomarkers, such as blood chitotriosidase and urinary glycosaminoglycans. The feasibility of these methods was evaluated, in order to consider them as a potential alternative to commercially available methods.

## Materials and Methods

### Materials

The following reagents 4-methylumbelliferyl acetate, 4-methyllumbelliferyl-α-D galactopyranoside, N-acetil-D-galactosamine, 4-methyllumbelliferyl-β-D-glucopyranoside, taurodeoxycholate hydrate, acarbose, 4-methyllumbelliferyl-α-D-glucopyranoside, 4-methylumbelliferylβ-D-N,N’,N’’triacetylchitotrioside, ethylenediamine dihydrochloride, formic acid, chondroitin sulfate sodium salt from shark cartilage, 1,9-dimethyl-methylene blue zinc chloride double salt, sodium hydroxide (NaOH) were purchased from Sigma-Aldrich (St. Louis, MO, USA). 4-methyllumbelliferyl sulfate potassium and 4-methyllumbelliferyl α-L-iduronide were purchased from Glycosynth (Warrington, UK). Sodium acetate, sodium citrate, sodium phosphate, and aminoacetic acid (glycine) were purchased from Synth (São Paulo, Brazil).

### Subjects

DBS samples of 834 newborns were collected at the Hospital da Pontifícia Universidade Católica (PUC) Campinas and Hospital Maternidade Celso Pierr, both in Campinas, SP, Brazil, after informed consent was obtained. Urine samples of 722 newborns were obtained from the same hospital, also after informed consent was obtained. Reference values for each of the analyzed enzymes for newborns were established using 131 de-identified DBS samples from healthy newborns, supplied by a commercial laboratory. As no positive samples of newborns were available, samples of patients diagnosed later in life (MPS I: 22, MPS VI: 24, Fabry: 12, Gaucher: 29, Pompe: 14) were used to establish the affected reference levels. All samples were shipped to the Medical Genetics Service (MGS) of Hospital de Clínicas de Porto Alegre (HCPA) and stored at -20 °C until the assays were conducted. This study was approved by the Institutional Review Board (IRB) from HCPA and from the Hospital da PUC-Campinas and Hospital Maternidade Celso Pierro (CAAE number: 2.824.531).

### Sample preparation

Enzyme assays were performed in a PerkinElmer incubator at 40 rpm. Fluorescence was measured in the supernatant by spectrofluorometry (Spectramax M2, Molecular Devices, San José, CA, USA) with 365 nm for excitation and 450 nm of emission. The results for 4-metilumbeliferone and enzyme activity were expressed as nmol/h/mL. All assays were done in duplicates.

### Alpha-L-iduronidase (IDUA) assay

One disk (1.5 mm) was cut from DBS samples and placed into a 96 well plate with 10 μL of deionized water (MilliQ, Millipore, Burlingto, MA, USA), 10 μL of 50 mM formate buffer (pH 2.8) and 7 μL of 2 mM 4-methyllumbelliferyl-α-L-iduronide substrate diluted in MilliQ water. In the blank wells, 240 μL of 0.5 M glycine-NaOH buffer (pH 10.3) were added. After mixing, the microplates were sealed and incubated per 20 h at 37 °C on a shaker (400 rpm). After incubation, the reactions were stopped by addition of 240 μL of 0.5 M glycine-NaOH buffer (pH 10.3). Samples were centrifuged at 2,500 rpm for 10 min at room temperature.

### Arylsulfatase B (ARSB) assay

One disk (1.5 mm) was cut from DBS samples and placed into a 96 well plate with 11.5 μL of MilliQ water, 7.5 μL of 15 mM lead acetate (pH 5) in 0.05 M of sodium acetate buffer (pH 5) and 18.75 μL of 10 mM 4-methylumbelliferyl-sulfate diluted in 0.05 M sodium acetate (pH 5). In the blank wells, 112.5 μL of 0.085 M glycine-NaOH buffer (pH 10.5) were added. After mixing, the microplates were sealed and incubated per 20 h at 37 °C on a shaker (400 rpm). After incubation, the reactions were stopped by addition of 112.5 μL of 0.085 M glycine-NaOH buffer (pH 10.5). Samples were centrifuged at 3,000 rpm for 10 min at 4 °C. 

### Alpha-galactosidase A (GLA) assay

One disk (1.5 mm) was cut from DBS samples and placed into a 96 well plate with 20 μL of 0.25 M N-acetyl-D-galactosamine inhibitor, 50 μL of 4-methylumbelliferyl-α-D-galactoside substrate diluted in 0.15 M citrate-phosphate buffer (pH 4.4). In the blank wells, 230 μL of 0.1 M ethylenediamine buffer (pH 11.4) were added. After mixing, the microplates were sealed and incubated per 20 h at 37 °C on a shaker (400 rpm). After incubation, the reactions were stopped by addition of 230 μL of 0.1 M ethylenediamine buffer (pH 11.4). Samples were centrifuged at 2,000 rpm for 5 min at room temperature.

### Beta-glucocerebrosidase (ABG) assay

One disk (1.5 mm) was cut from DBS samples and placed into a 96 well plate with 12.5 μL of 0.54 M citrate-phosphate buffer (pH 5.5), 25 μL of 10 mM 4-methylumbelliferyl-β-D-glucoside substrate and 50 mM sodium taurodeoxycholate diluted in MilliQ water. In the blank wells, 250 μL of 0.5 M glycine-NaOH buffer (pH 10.3) were added. After mixing, the microplates were sealed and incubated per 5h at 37 °C on a shaker (400 rpm). After incubation, the reactions were stopped by addition of 250 μL of 0.5 M glycine-NaOH buffer (pH 10.3). Samples were centrifuged at 3,000 rpm for 10 min at room temperature.

### Acid alpha-glucosidase (GAA) assay

One disk (1.5 mm) was cut from DBS samples and placed into a 96 well plate with 10 μL of MilliQ water, 20 μL of 10 mM 4-methylumbelliferyl-α-D-glucoside substrate diluted in 0.2 M citrate-phosphate buffer (pH 4) and 8 mM acarbose diluted in MilliQ water. In the blank wells, 240 μL of 0.5 M glycine-NaOH buffer (pH 10.3) were added. After mixing, the microplates were sealed and incubated per 20 h at 37 °C with shaker (400 rpm). After incubation, the reactions were stopped by addition of 240 μL of 0.5 M glycine-NaOH buffer (pH 10.3). Samples were centrifuged at 2,000 rpm for 5 min at room temperature.

### Chitotriosidase assay

One disk (1.5 mm) was cut from DBS samples and placed into a 96 well plate with 10 μL of 0.25 M sodium acetate (pH 5.5), 10 μL of 0.19 nM 4-methylumbelliferyl-β-D-N-N’-N’’-triaceltylchitotrioside substrate diluted in MilliQ water. In the blank wells, 250 μL of 0.1 M ethylenediamine buffer (pH 11.3). After mixing, the microplates were sealed and incubated per 30 min at 37 °C on a shaker (400 rpm). After incubation, the reaction was stopped by addition of 250 μL of 0.1 M ethylenediamine buffer (pH 11.3). Samples were centrifuged at 3,000 rpm per 10 min at room temperature.

### Glycosaminoglycans assay

Urine samples were centrifuged at 2,000 rpm for 10 min. Fifty microliters of sample were mixed with 1.1 mL of buffer (55 mM formic acid buffer, pH 3.3) with 31 μM of dimethylmethylene-blue and 2 M tris(hydroxymethyl)aminomethane. After vortexing, absorbance was immediately measured in a spectrophotometer at 520 nm. Chondroitin sulfate was used for a calibration curve at the following concentrations: 50 mg/L, 25 mg/L, 12.5 mg/L and 5 mg/L. Urinary GAGs were normalized by creatinine, and concentrations were expressed as μg/mg creatinine.

### Statistical analysis

Sensitivity, specificity, false positives and false negatives were calculated using R software (R Core Team, 2014). Potential cutoff values for the five enzymes were defined using normal newborn percentile obtained from 131 healthy newborns and enzyme level from older patients provided by our laboratory. Enzyme activities for IDUA, ARSB, GLA and GBA was expressed as nmol/20h/mL; enzyme activity of ABG were expressed as nmol/5h/mL; glycosaminoglycan concentration was expressed as μg/mg/creatinine; and chitotriosidase activity was expressed as nmol/h/mL.

## Results

The major adaptations in the current methods for these enzyme assays were: reduction of the original reaction volume (substrate/reagents) and reduction of the DBS punch from 3 mm to 1.5 mm (Chamoles *et al.*, 2001; Civallero *et al.*, 2006). This led to a cost reduction compared to the original method described by Chamoles *et al.* (2001).

### Sensitivity and specificity

Sensitivity, specificity, positive and negative predictive values for all enzymes are described in Table 1.

**Table 1:** Sensitivity, specificity, positive predictive values, and negative predictive values for the five analyzed enzymes.

Enzyme	Sensitivity	Specificity	PPV	NPV
*IDUA*	100%	94%	32%	100%
*ARSB*	100%	99.7%	89%	100%
*GLA*	100%	99.9%	93%	100%
*ABG*	100%	98%	67%	100%
*GAA*	100%	97%	36%	100%

IDUA: alpha-L-iduronidase; ARSB: arylsulfatase B; GLA: alpha-galactosidase A; ABG: beta-glucocerebrosidase; GAA: acid alpha-glucosidase; PPV: positive predictive values; NPV: negative predictive values

### Determination of normal and affected ranges

In order to define the normal range for each enzyme, DBS from 131 healthy de-identified newborns were analyzed and reference values are described in Table 2. To establish potential disease cutoff values, samples of known patients were analyzed for MPS I (22), MPS VI (24), Fabry (12), Gaucher (29), and Pompe (14) (Table 2).

**Table 2:** Normal and affected ranges for each enzyme.

Enzyme	Reference range for healthy newborns (n=131)	Range of affected patients
*IDUA*	0.8-4*	0-0.5*
*ARSB*	3-16*	0.9-2.4*
*GLA*	4-21*	0.4-1.1*
*ABG*	2-10**	0.1-1.6**
*GAA*	12-63*	0-5.5*
*Chitotriosidase*	0-53#	166-677#

IDUA: alpha-L-iduronidase; ARSB: arylsulfatase B; GLA: alpha-galactosidase A; ABG: beta-glucocerebrosidase; GAA: acid alpha-glucosidase; * nmol/20h/mL; ** nmol/5h/mL; # nmol/h/ml.

### Determination of normal and affected ranges

*Cutoff values*

**Figure 1:**
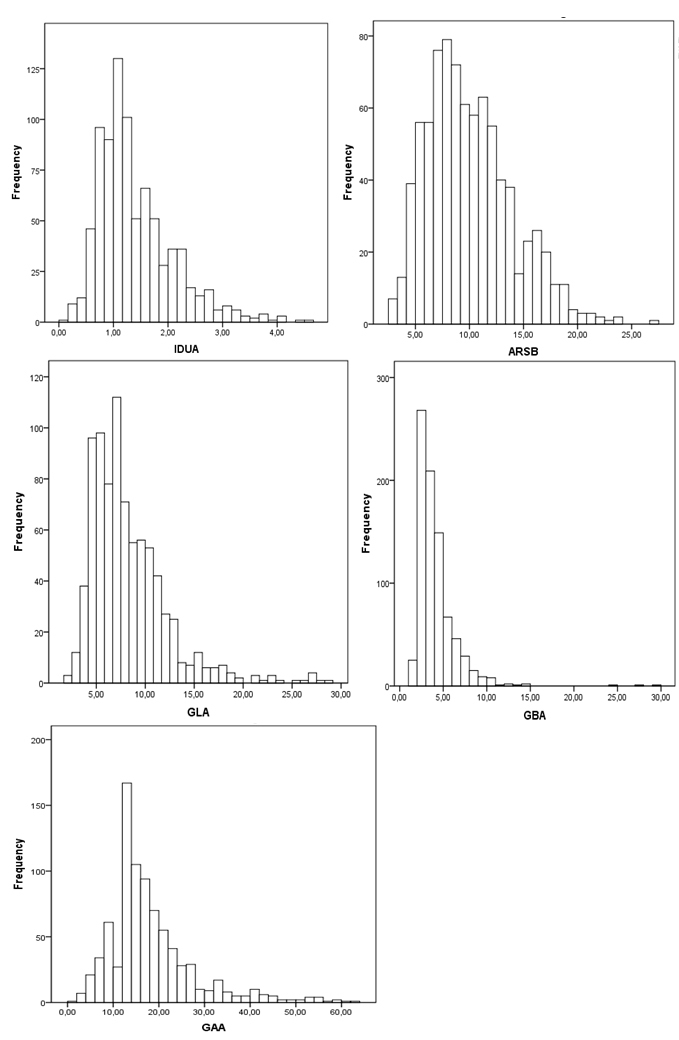
Distribution of the five enzymes analyzed in this study by fluorimetry. IDUA: alpha-L-iduronidase; ARSB: arylsulfatase B; GLA: alpha-galactosidase A; ABG: beta-glucocerebrosidase; GAA: acid alpha-glucosidase.

In this study, 834 DBS samples of newborns were analyzed. The distributions of the five enzymes are shown in Figure 1. Initial cutoffs were set as values below the reference values established in healthy de-identified newborns (IDUA < 0.8 nmol/20h/mL; ARSB < 3 nmol/20h/mL; GLA< 4 nmol/20h/mL; ABG < 12 nmol/5h/mL and GAA < 2 nmol/20h/mL) (Table 2). Using these reference values, the false positive rates were: 10.5% for IDUA (n=88), 0.8% for ARSB (n=3), 3.7% for GLA (n=31), 3% for ABG (n=25), and 18% for GAA (n=150).

In order to decrease the false positive rates, we decided to use the highest enzyme level of known patients. With this we aimed at avoiding any false negative, once all of these were lower than the lowest value of the reference range for healthy newborns (Table 2). Thus, false positive rates decreased to: 5.5% for IDUA (n=46), 0.8% for ARSB, 0% for GLA, 1.7% for ABG (n=14), and 3% for GAA (n=25).

For MPS I, MPS VI, and Gaucher disease we also used a second-tier test in order to further decrease the false positive rates. For MPS I and VI we used urinary GAGs as a biomarker, and for Gaucher disease chitotriosidase in DBS. For MPS I, the original false positive rates were 5.5% (n=46) using reference range, and of these 76% (n=35) had urine samples for GAG measurement all within normal levels (reference range up to 6 months: 133-460 μg/mg/creatinine). Thus, false positive rates decreased even further to 1.3%. For the three MPS VI cases with low ARSB levels, two had urine available for GAG measurement, with normal GAG levels resulting in 0.1% false positives. For Gaucher disease, we used the measurement of chitotriosidase to reduce the false positive rates. Of the 1.7% (n=14), only one had elevated levels of chitotriosidase (62, reference range: 0-53 nmol/h/mL), reducing the false positive rate to 0.1%.

All samples with abnormal enzyme levels will be further investigated by the measurement of enzyme activity in leukocytes, as well as gene sequencing to exclude any abnormality.

## Discussion

Newborn screening for lysosomal diseases has been performed regularly since 2006 for Krabbe disease, in the state of New York, USA (Orsini *et al.*, 2016), and is being included in the newborn screening panels by several states in the USA (Hopkins *et al.*, 2015; Burton *et al.*, 2017), being already an official recommendation for MPS I and Pompe (Minter Baerg *et al.*, 2018). It is also a regular practice in Taiwan (Chuang *et al.*, 2018) and in some regions of Italy (Burlina *et al.*, 2018). All the efforts to include LSDs to NBS are due to the lack of clinical signs at birth, the progressive characteristic of these disorder, and availability of treatment that seems to be more effective when started early (McGill *et al.*, 2010; Giugliani *et al.*, 2010).

Newborn screening for lysosomal storage disorders can be done by fluorometry or tandem mass spectrometry (MS/MS). The superior analytical range of MS/MS compared to fluorimetric assays has already been described by several groups (Gelb *et al.*, 2015; Kumar *et al.*, 2015; Schielen *et al.*, 2017). However, a digital microfluid method has been used by several centers with optimized cut-offs to decrease false positive rates (Hopkins *et al.*, 2015).

This study has several limitations, such as the lack of newborn samples of known patients to determine and validate the cutoffs, the inability to multiplex the five substrates due to the same excitation/emission wavelength for all enzymes, and the fact that all the reagents were made “in-house”, without the manufacturing standardization of commercially available kits.

For MPS I, MPS VI and Gaucher disease we were able to reduce the false positive rates using a second-tier approach throughout biomarker measurement (glycosaminoglycans for MPS and chitotriosidase for Gaucher). Nonetheless, for Pompe disease we had false positives rates that were higher compared to the other disorders. This could have been caused by the fact that the acid alpha-glucosidase (GAA) substrate preparation requires a much higher temperature (50-60 °C), which could potentially affect the enzyme reaction. Another technique that produces lower false positive rates has been described for detection of Pompe disease (communication from LEIM-HCPA). However, more steps in the sample preparation are required, as well as higher amounts of sample, which could potentially make it unsuitable for mass screening.

We conclude that these optimized methods are efficient and feasible for detection of five LSDs in NBS. These new methods allowed a reduction of 50% of the original cost (from US$ 60.00 to US$ 30.00 per sample, for all the assays) which could potentially enable their adoption for the screening of LSDs in lower-income countries, or in centers that do not have access to tandem mass spectrometry.
